# The Posterior Dislocation of the Shoulder With Reverse Hill-Sachs Lesion and Humerus Lesser and Greater Tuberosity Fracture

**DOI:** 10.7759/cureus.65333

**Published:** 2024-07-25

**Authors:** Ishan Shevate, Vikram Reddy Cheemala, Ashwin Deshmukh, Rahul Salunkhe

**Affiliations:** 1 Orthopedics and Trauma, Dr. D. Y. Patil Medical College, Hospital & Research Centre, Pune, IND

**Keywords:** proximal humerus, rotator cuff tears, case report, greater tuberosity fracture, mclaughlin technique, epileptic seizures, glenoid, shoulder trauma, humeral tuberosity fracture, reverse hill-sachs lesion

## Abstract

Posterior shoulder dislocations are the rarest of all shoulder dislocations. They are commonly associated with seizures, electric shocks, or trauma. This case report presents a 60-year-old male with a posterior shoulder dislocation complicated by fractures of the greater tuberosity (GT) and lesser tuberosity (LT) and a reverse Hill-Sachs lesion. The patient was treated surgically using a modified McLaughlin procedure. This case highlights the importance of the early recognition and appropriate surgical management of complex posterior shoulder dislocations to prevent recurrent instability and ensure optimal functional recovery.

## Introduction

In all shoulder dislocations, posterior dislocation is found in only 2%-5% [1]. Seizures, electric shock, and direct or indirect trauma that occurs with the flexion, adduction, and internal rotation of the shoulder are the major causes of posterior dislocations [2]. Posterior dislocations are sometimes associated with surgical neck fractures or fractures of the tuberosity. Most stable posterior dislocations with no significant bone defect are treated by conservative management. If a bone defect exists, depending on size and location, the reverse Hill-Sachs lesion leads to relocation and often requires surgical management [3]. The diagnosis is usually missed during the initial assessment, despite its typical mechanisms of injury, clinical presentation, and radiological signs [4-6]. A reverse Hill-Sachs lesion, a subchondral fracture, or a large lesser tuberosity (LT) fracture is frequently present in posteriorly locked shoulder dislocations. This lesion might cause significant early joint damage and osteoarthritis (OA) [7]. Locked dislocations should be reduced under anesthesia by closed reduction most often, but an open procedure also might be required at times [5]. Various surgical managements have been described for the management of reverse Hill-Sachs lesions, such as the medial transposition of the lesser tuberosity [8], defect-filling with autograft or allograft [9], posterior bone block [10], derotational osteotomy [11], and arthroscopic or open remplissage with the subscapularis tendon [12]. The McLaughlin technique is the most common surgical procedure performed for a reverse Hill-Sachs lesion. McLaughlin first described it in 1952, when the subscapularis tendon detached from its origin and was inserted into the bare area of the humerus [8]. Hawkins et al. (1987) modified the technique by performing an osseous transfer of the subscapularis tendon insertion and the lesser tuberosity into the defect [13]. In the present case, a combination of posterior dislocation with a greater tuberosity (GT) fracture, a lesser tuberosity fracture, and a reverse Hill-Sachs lesion is present, with only very few similar cases being reported [14-16].

## Case presentation

**Figure 1:**
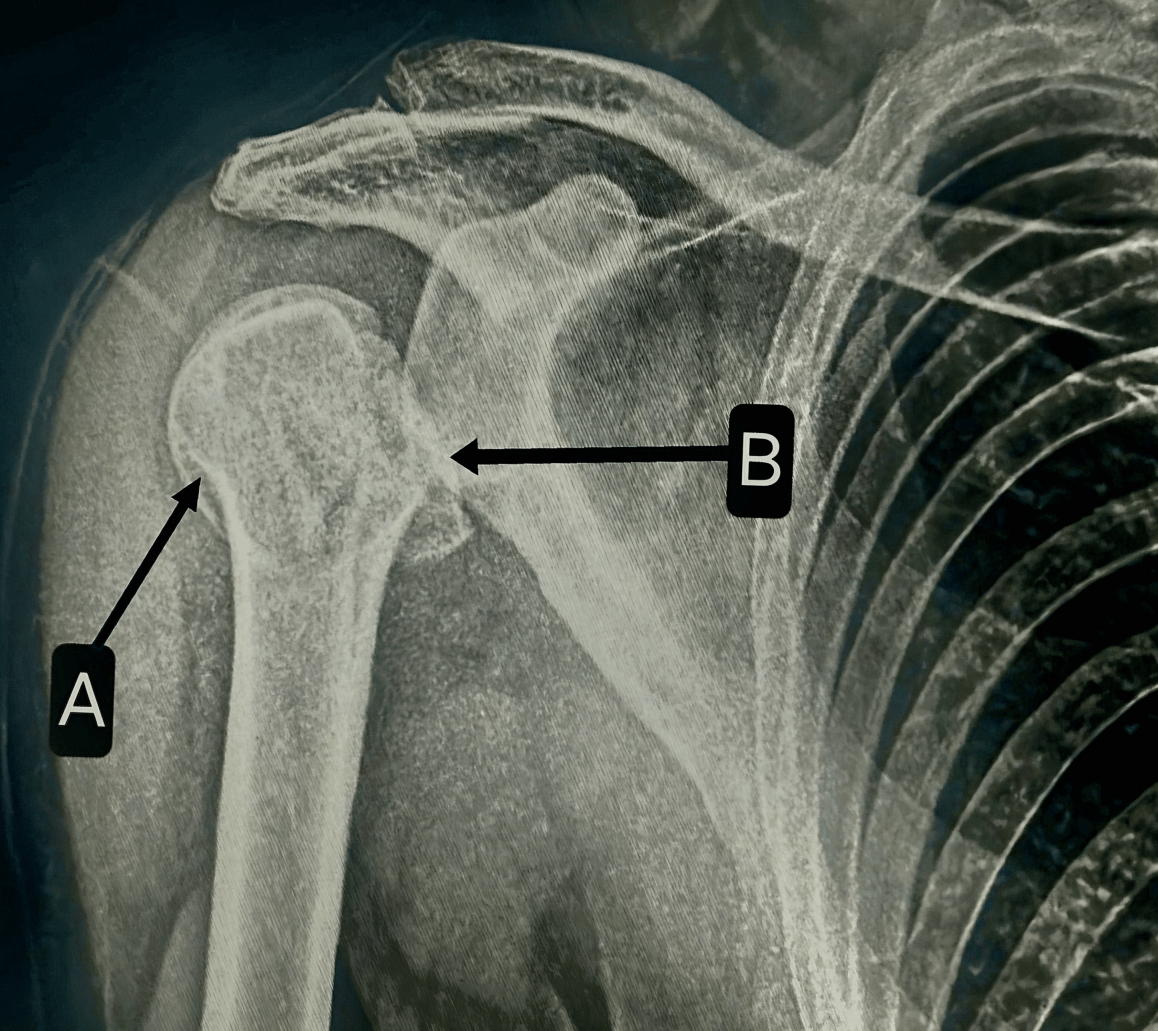
X-ray of the right shoulder showing GT fracture (A) and LT fracture (B) with a posterior dislocation of the shoulder. GT, greater tuberosity; LT, lesser tuberosity

**Figure 2:**
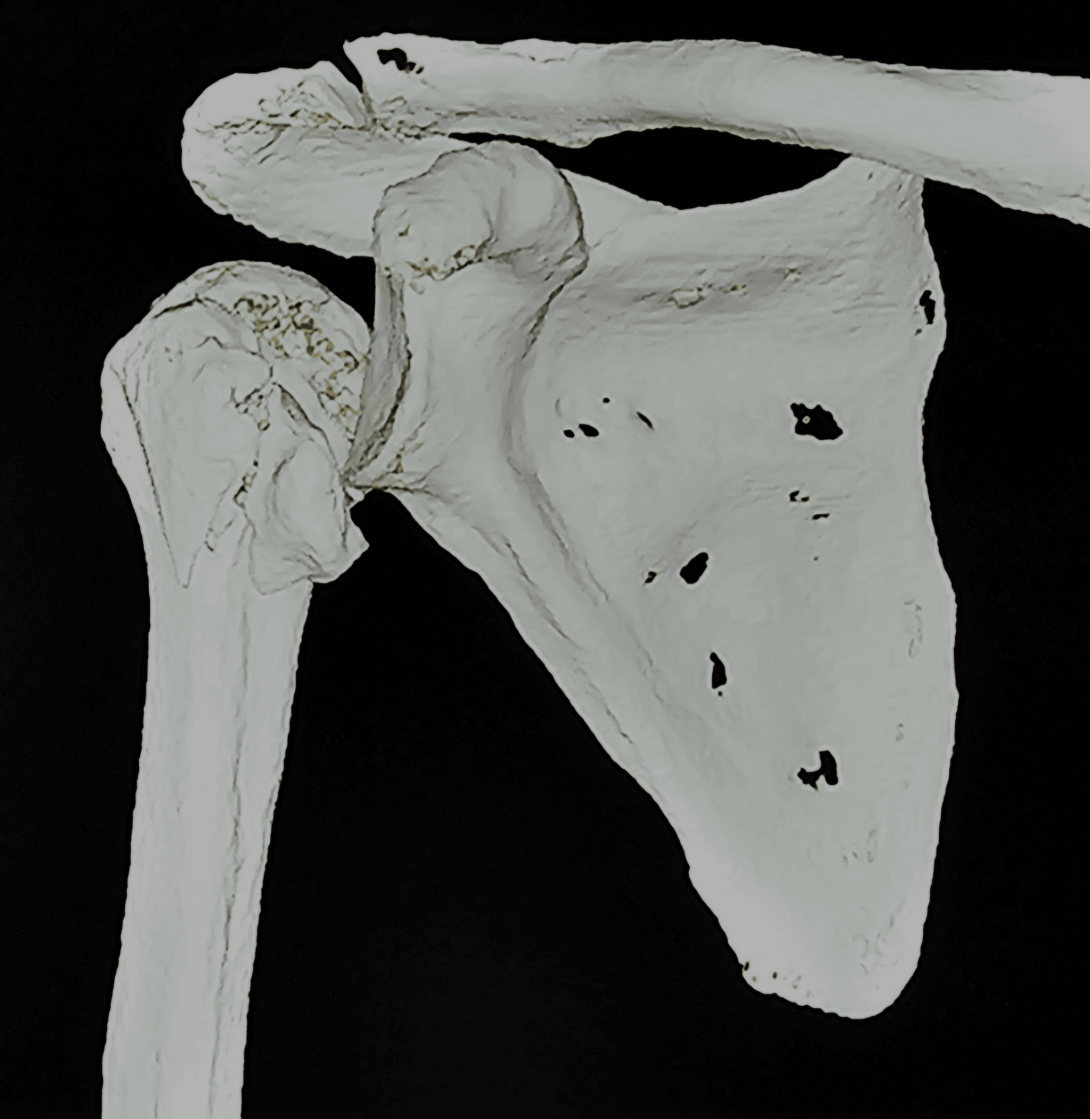
CT images with reverse Hill-Sachs lesion with GT and LT fractures. CT, computed tomography; GT, greater tuberosity; LT, lesser tuberosity

**Figure 3:**
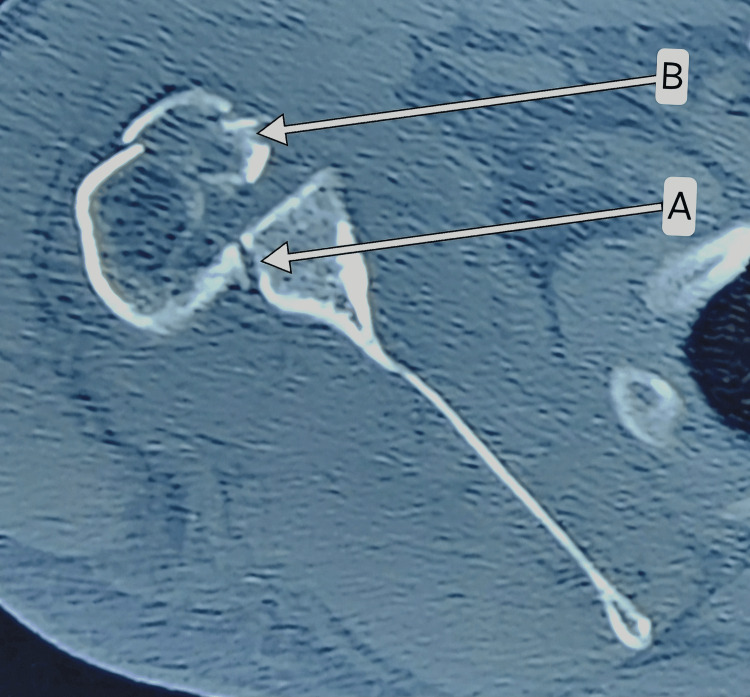
Axial plane CT image showing reverse Hill-Sachs lesion (A) with GT and LT fractures (B). CT, computed tomography; GT, greater tuberosity; LT, lesser tuberosity

**Figure 4:**
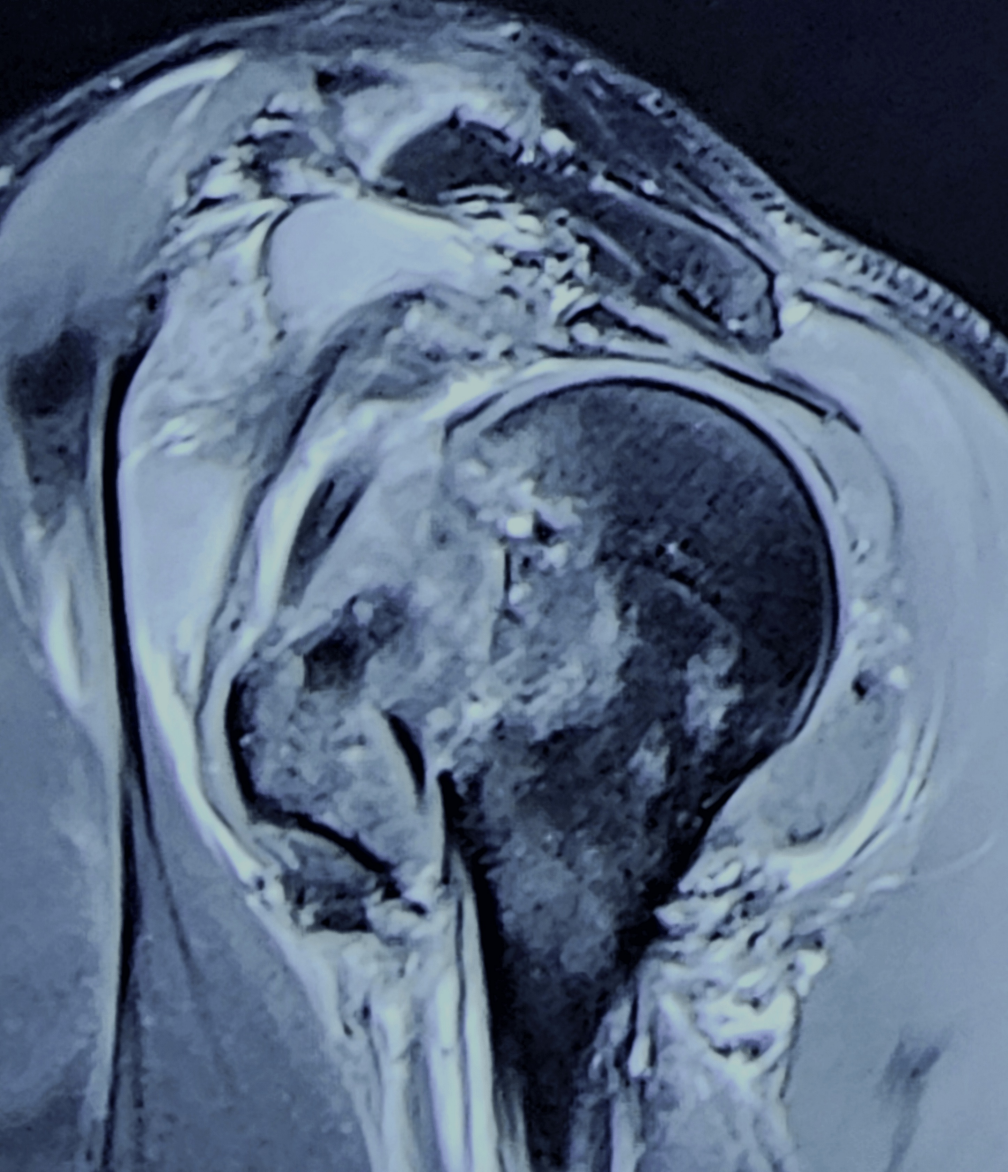
MRI (sagittal view) showing GT fracture with a subluxated long head of biceps tendon from the bicipital groove. MRI, magnetic resonance imaging; GT, greater tuberosity

A 60-year-old male patient was on regular anti-epileptic medication (tablet phenytoin 300 mg once daily {OD}) and presented to the emergency department with one episode of seizure the day before the admission with complaints of pain in the right shoulder and inability to perform movements in the right shoulder. The patient examined tenderness noted around the right shoulder, and all range of movements were restricted and associated with pain; the empty glenoid sign and the Hamilton ruler test were positive. On diagnostic imaging, the patient was diagnosed with a posterior dislocation of the right shoulder with greater tuberosity (GT) and lesser tuberosity (LT) fractures, along with a reverse Hill-Sachs lesion (Figures 1-4). The shoulder joint was unstable on the reduction of the dislocation. The patient was posted for open shoulder reduction with the modified McLaughlin procedure and GT fixation. The patient was under general anesthesia in the beach chair position, and the shoulder joint was exposed by the deltopectoral approach. The greater and lesser tuberosity fractures were noted, along with the subluxation of the long head of the biceps tendon from the bicipital groove.

**Figure 5:**
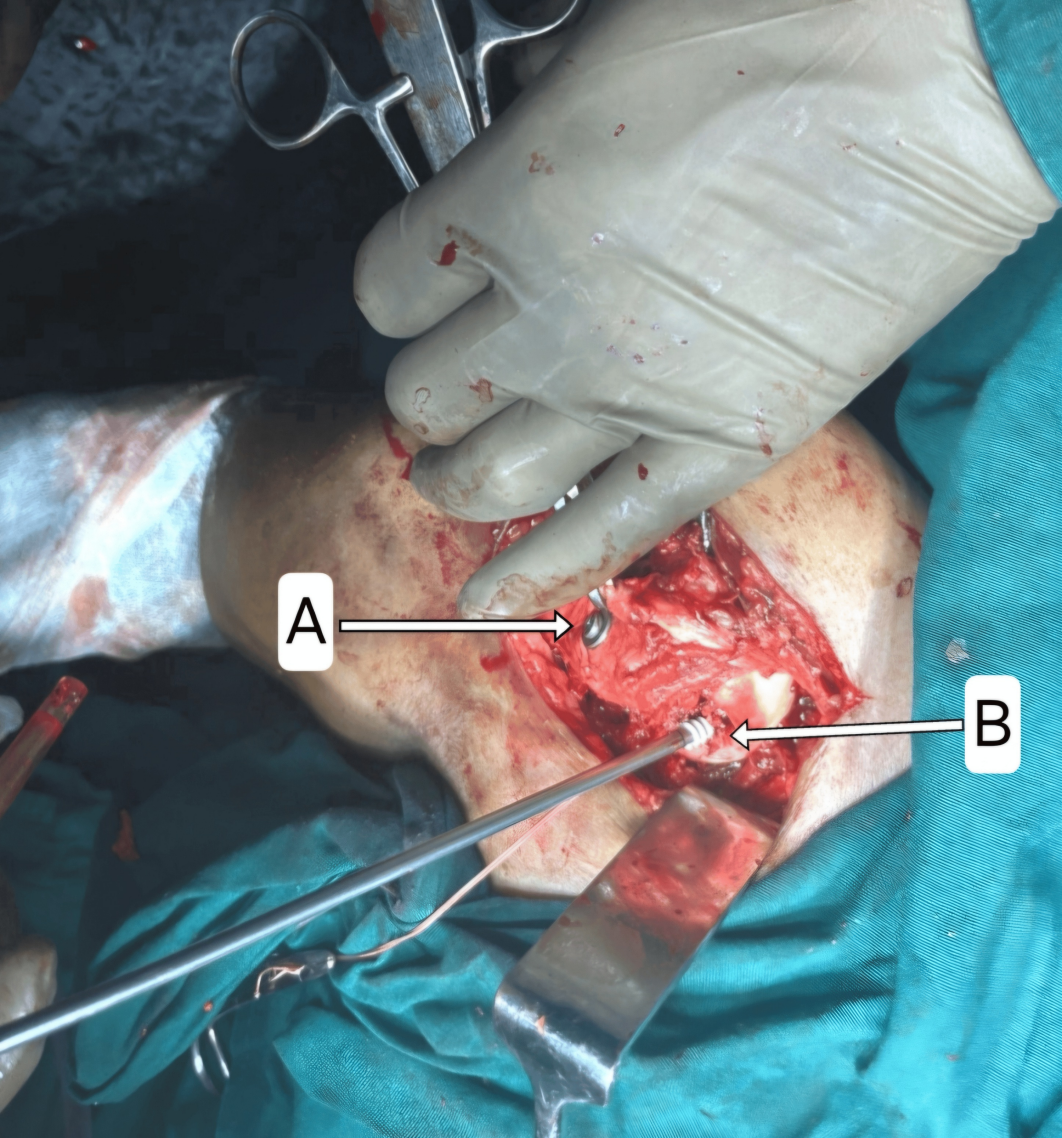
Open reduction of GT fracture and fixation with CC screw (A) and the placement of double-loaded 2.3 mm PEEK anchors (B) into the bare area of the anteromedial humerus head. GT, greater tuberosity; CC, corticocancellous; PEEK, polyetheretherketone

**Figure 6:**
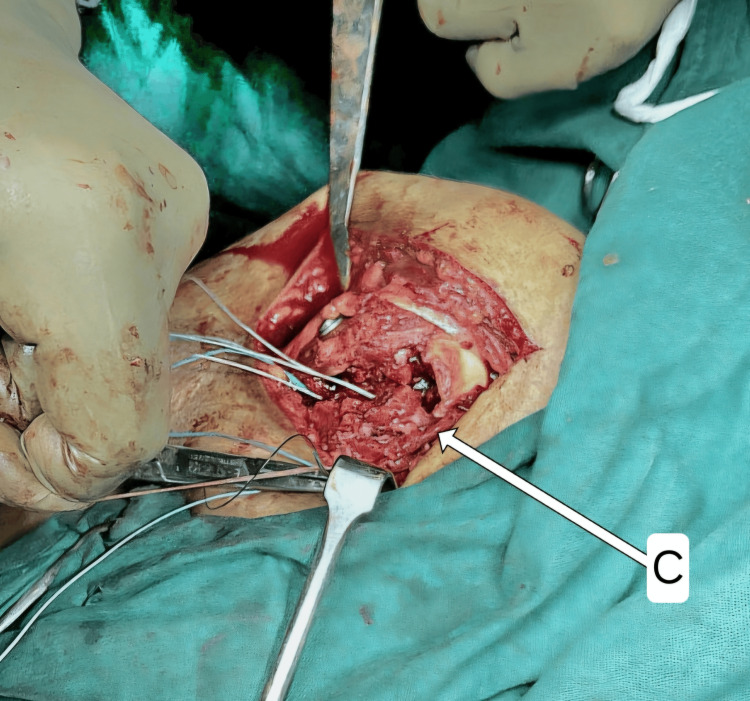
Medial limbs of sutures passed transosseous around the LT (C) LT: lesser tuberosity

**Figure 7:**
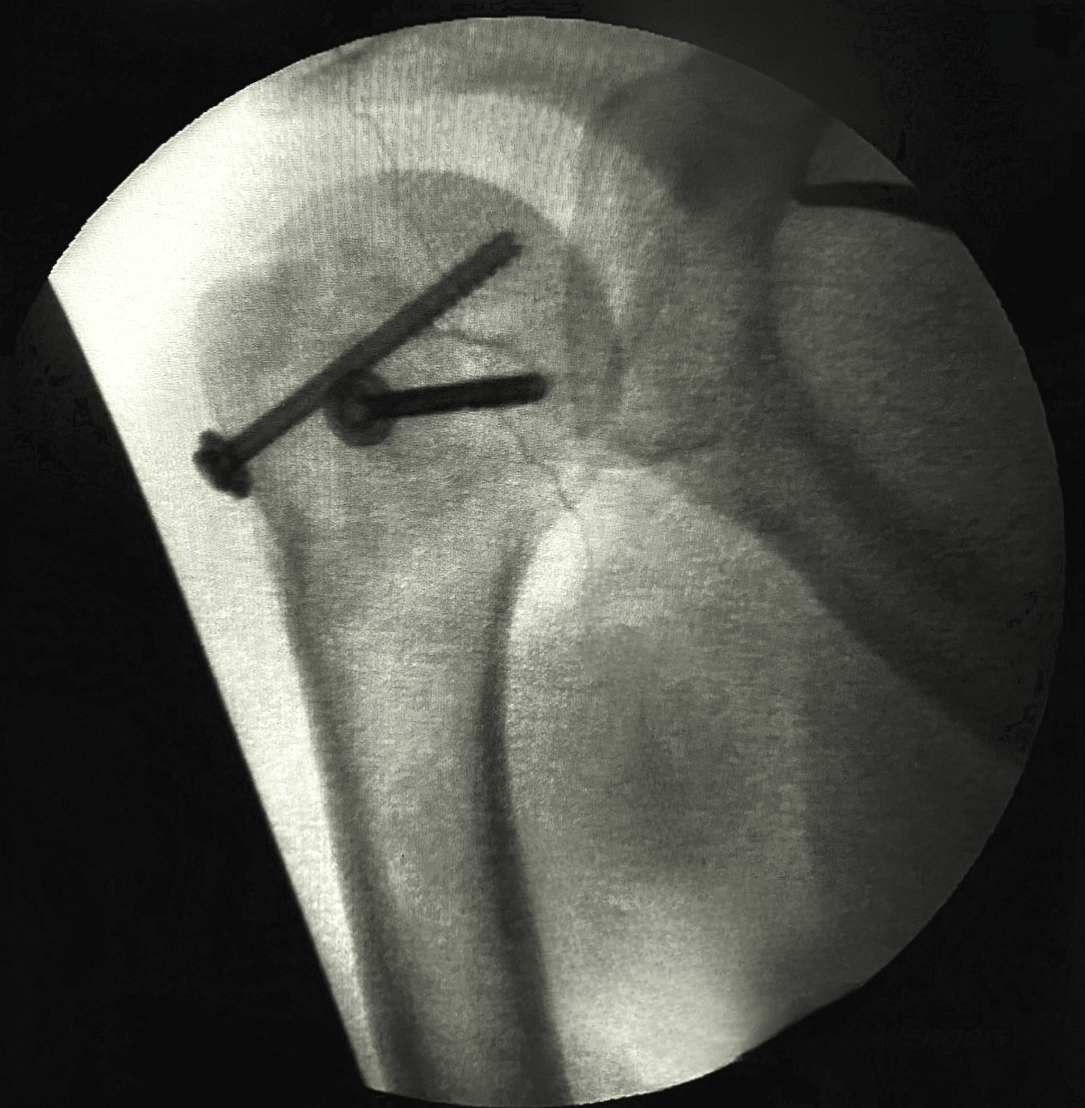
Intraoperative fluoroscopic image of the right shoulder showing reduced shoulder dislocation with GT fracture fixation and modified McLaughlin procedure for reverse Hill-Sachs lesion GT: greater tuberosity

By gentle traction and manipulation, the humeral head was reduced to its original position, and the reverse Hill-Sachs lesion was identified. The fractured lesser tuberosity was placed over the bare area of the anterior humeral head, and two 5.5 mm polyetheretherketone (PEEK) suture anchors were placed superiorly and inferiorly; sutures passed through the lesser tuberosity (Figures 5, 6). Sutures were passed in a mattress fashion, transfixed LT to the bare area, and were reinforced with a 4.5 mm titanium corticocancellous screw (Figures 6, 7). The greater tuberosity was reduced and fixed with a single 4.5 mm corticocancellous screw (Figure 7). The long head of the biceps tendon was relocated into the bicipital groove, and stability was assessed in all ranges of movements. The patient was immobilized in a 30-degree shoulder splint. After one month, the graded mobilization of the shoulder was started, and over the next three months, the patient developed full-range movements with no further episodes of dislocation. The patient started his daily activities and regular work with not much difficulty. The radiographic imaging showed satisfactory healing of the greater and lesser tuberosities with no signs of instability on clinical examination.

## Discussion

Posterior shoulder dislocation is a rare entity, of which only 0.9% represents posterior fracture-dislocation [16]. In fracture-dislocation cases, the patients presented with humeral neck fractures in 55%, LT fractures in 42%, and GT fractures in 23% [7,17]. The presence of bony and subchondral lesions in posterior dislocations is noted in 65% of the cases [17]. Twenty-nine percent presents with a fracture of the anteromedial portion of the humeral head, with an increased incidence with age. The mechanism of injury in posterior dislocation is unbalanced muscle contractions, i.e., electric shock and epileptic seizures [15]. In seizures, it is more common as the contractions of the comparatively weak teres minor and infraspinatus and the posterior fibers of the deltoid are overcome by more powerful internal rotators, i.e., the subscapularis, latissimus dorsi, and anterior fibers of the deltoid, resulting in the internal rotation and posterior subluxation of the shoulder [18].

Various surgeries have been described for different fracture patterns in posterior fracture-dislocation. For anteromedial fractures and humeral head defects of >25%, the modified McLaughlin procedure and autograft/allograft bone grafting are performed [8,13]. The most common fracture sites are the humeral neck and the lesser tuberosity in posterior dislocations. Ogawa et al., in a series of four cases, suggested conservative treatment in spontaneous fracture reduction and internal fixation in fragment displacement greater than 10 mm [19]. Hayes et al. reported a case study of posterior dislocation with LT fracture treated by internal fixation and conserved cases with spontaneous fragment reduction without displacement. He stated that by the internal fixation of the fracture, the patients can be mobilized at the earliest and start rehabilitation [20]. A greater tuberosity fracture is a rare injury in the case of a posterior fracture-dislocation. Mutchamee and Pongsamakthai, in a case report, stated that a posterior dislocation along with a GT fracture was treated with a closed reduction [13]. Zufahrizzat et al. reported a GT fracture along with a posterior dislocation, treated by internal reduction with partially threaded screws [16]. Various cases were reported with posterior dislocation with isolated GT fractures, LT fractures, and reverse Hill-Sachs lesions. Kotsalis et al. reported a case with both GT and LT fractures managed by internal fixation with the proximal humerus locking system (PHILOS) and suture anchors, respectively [7]. In the present case, both GT and LT were fractured with a reverse Hill-Sachs lesion, which was managed by GT fixation using a partially threaded screw and modified McLaughlin procedure, respectively.

## Conclusions

This case underscores the importance of recognizing and appropriately managing complex shoulder injuries, such as reverse Hill-Sachs lesions with associated humeral tuberosity fractures. A multidisciplinary approach is essential for achieving favorable outcomes and minimizing the risk of recurrent instability. Further studies are warranted to elucidate optimal treatment strategies for these rare and challenging injuries.
